# Performance of PEth Compared With Other Alcohol Biomarkers in Subjects Presenting For Occupational and Pre-Employment Medical Examination

**DOI:** 10.1093/alcalc/agaa027

**Published:** 2020-05-04

**Authors:** Jasna Neumann, Olof Beck, Anders Helander, Michael Böttcher

**Affiliations:** 1 MVZ Labor Dessau GmbH, Bauhüttenstrasse 6, D-06847 Dessau-Roßlau, Germany; 2 Department of Clinical Neuroscience, CPF, Norra Stationsgatan 69, Karolinska Institutet, Stockholm, SE-171 77, Sweden; 3 Department of Laboratory Medicine, C1:74, Karolinska University Hospital, Karolinska Institutet, Stockholm, SE-141 86, Sweden

## Abstract

**Aims:**

To compare the performance of short- and long-term alcohol biomarkers for the evaluation of alcohol drinking in employment-related health controls.

**Methods:**

The 519 blood samples originated from 509 patients (80% men) presenting at occupational health units and medical centers at employment agencies for the evaluation of risky drinking. The laboratory investigation comprised the measurement of phosphatidylethanol (PEth 16:0/18:1), carbohydrate-deficient transferrin (CDT; % disialotransferrin), gamma-glutamyl transferase (GGT), mean corpuscular volume (MCV), ethanol and ethyl glucuronide (EtG).

**Results:**

Many samples tested positive for acute (57%) and chronic (69%) alcohol biomarkers. PEth was the single most positive biomarker (64%; cut-off 0.05 μmol/l or 35 μg/l) and the only positive chronic biomarker in 100 cases. The highest PEth concentrations were seen in samples positive for all chronic biomarkers, followed by those also being CDT positive (cut-off 2.0%). All 126 CDT-positive samples were positive for PEth using the lower reporting limit (≥0.05 μmol/l) and for 114 cases (90%) also using the higher limit (≥0.30 μmol/l or 210 μg/l). In the CDT-positive cases, the PEth median concentration was 1.71 μmol/l, compared with 0.45 μmol/l for the CDT-negative cases (*P* < 0.0001). PEth and CDT values were correlated significantly (*r* = 0.63, *P* < 0.0001). Among the EtG-positive cases (≥1.0 ng/ml), 95% were also PEth positive, and all ethanol-positive cases (≥0.10 g/l) were also PEth positive.

**Conclusions:**

For optimal detection of drinking habits, using a combination of short- and long-term alcohol biomarkers provided best information. PEth was the single most positive alcohol biomarker, whereas GGT and MCV offered little additional value over PEth and CDT.

## Teaser

Short summary Phosphatidylethanol (PEth) was the most often alcohol biomarker. Carbohydrate-deficient transferrin and PEth responded differently to heavy alcohol drinking, and gamma-glutamyl transferase and mean corpuscular volume offered no additional value. Most cases positive for ethanol and EtG were also PEth positive. A combination of short- and long-term alcohol biomarkers provided best information regarding drinking habits.

## INTRODUCTION

Alcohol consumption is associated with an elevated risk for serious health problems; it affects a large proportion of the population globally and results in significant economic consequences (Rehm *et al.*, 2018). The health risks are related to the daily amount of alcohol intake (Corrao *et al.*, 2004), and the lower threshold for harmful drinking was recently proposed to be 100 g/week (Wood *et al.*, 2018). WHO has pointed out the implementation of screening for harmful drinking and brief intervention programs as recommended actions, to reduce the burden of excessive alcohol consumption (WHO, 2018).

For screening of harmful alcohol use, alcohol biomarkers are important objective complements to self-reports and structured interviews about drinking habits and can be used in health controls to aid early detection (Hermansson *et al*., 2010). Other applications of alcohol biomarkers are to monitor compliance in treatment, to monitor drinking during pregnancy and in subjects applying for job, or in regranting of a driving license (Isaksson *et al.*, 2011).

A range of alcohol biomarkers are available based on measurement in blood, urine or hair samples (Niemelä, 2016). A recently introduced biomarker is phosphatidylethanol (PEth) that is measured in whole blood by liquid chromatography-mass spectrometry (LC-MS) methods (Nalesso *et al.*, 2011; Zheng *et al.*, 2011). PEth has demonstrated superior specificity and sensitivity compared with other biomarkers targeting regular high alcohol use (‘chronic’ biomarkers) (Viel *et al.*, 2012). PEth is formed during ethanol exposure and accumulates over time following repeated and frequent drinking. Each intake of an intoxicating ethanol dose leads to an elevation of the PEth level in whole blood (Schröck *et al.*, 2017). During abstinence, the half-life of PEth differs between individuals in the range 4–10 (median 6) days (Helander *et al.*, 2019a).

PEth has recently been introduced as a routine alcohol biomarker to confirm abstinence or detect harmful drinking (Ulwelling and Smith, 2018). However, an important issue for the latter application is that it appears to be difficult to establish the magnitude of alcohol intake based on a PEth value, as several studies have demonstrated considerable variation in test response between different individuals (Aradottir *et al.*, 2006; Helander *et al.*, 2012, 2019b; Viel *et al.*, 2012; Ulwelling and Smith, 2018). Another important feature that needs additional investigation is the comparison of PEth with other alcohol biomarkers, and how they can best be used in combination. A recent comparison of PEth with other alcohol biomarkers and self-reports in pre- and post-liver transplant patients supported the superior features of PEth (Andresen-Streichert *et al*., 2017).

The aim of this work was to compare test results for a set of alcohol biomarkers commonly used to indicate recent drinking (called ‘acute’ biomarkers) or mainly regular high alcohol consumption (called ‘chronic’ biomarkers) in blood samples received for routine measurement from employment-related health controls.

## MATERIALS AND METHODS

### Clinical samples

The samples investigated in this study came from patients presenting at Medical Centers at employment agencies and from occupational health units. The subjects typically underwent evaluation concerning risky drinking and were predominantly unemployed. The study included 519 sample sets from 509 patients (80% were men and 8 gave repeated samples). EDTA-whole blood and serum samples taken by venous puncture were received for routine measurement of ethanol, carbohydrate-deficient transferrin (CDT, % disialotransferrin) and gamma-glutamyl transferase (GGT) in serum, and erythrocyte mean corpuscular volume (MCV) in whole blood. Left over aliquots of whole blood were stored at 4°C and used for ethyl glucuronide (EtG) and PEth analysis within 1 week after samples were anonymized.

### Analysis of PEth in whole blood

PEth analysis (PEth 16:0/18:1 form) was made by an in-house DIN EN ISO 17025 accredited UPLC-MS/MS method. Twenty microlitre of whole blood were mixed with 20-μl internal standard (0.142 μmol/l PEth-d_5_, Chiron AS, Trondheim, Norway) in isopropanol. To precipitate proteins, 120-μl isopropanol was added followed by mixing and addition of 200-μl *n*-hexane. After another mixing and centrifugation, the entire supernatant was transferred to new tubes and evaporated to dryness at 45°C for about 10 min with nitrogen. The dry residue was re-dissolved in 200 μl of a 1 + 4 mix of mobile phase A and B (see below). Following mixing and centrifugation, 10 μl was injected into the UPLC system. Calibration was performed using fortified blank blood at 0.014, 0.043, 0.085, 0.142, 0.356, 0.711, 1.14, 1.42, 3.56 and 7.11 μmol/l PEth 16:0/18:1 using reference material from Avanti Polar Lipids Inc. (Alabaster, AL, USA). For quality controls (QC) at a concentration of 0.05 μmol/l, the CV was 11% and bias 5.0%; at 0.30 μmol/l, the CV was 10.5% and bias 0.2% and at 2.85 μmol/l, the CV was 9.0% and bias 0%. Accuracy in the measurements was confirmed by participation in the EQUALIS AB (Uppsala, Sweden) quality assessment program. The lower quantification limit (LLOQ) was 0.014 μmol/l.

Chromatographic separation was conducted using a Waters Acquity UPLC connected to a Waters Xevo-TQ-S on a BEH C18 column (1.7 μm, 2.1 × 150 mm, Waters) within 3.5 min using gradient elution (mobile phase A = 5 mmol/l ammonium formate in water/isopropanol/acetonitrile (30/10/60; v/v/v), mobile phase B = 5 mmol/l ammonium formate in water/isopropanol/acetonitrile (1/79/20; v/v/v)). The analytical column was kept at 40°C and the flow rate of 0.30 ml/min. Capillary voltage was set to 1.5 kV, the ion source temperature was 150°C and desolvation gas was heated to 650°C and delivered at a flow rate of 800 l/h. Cone gas (N_2_) was set to 150 l/h, and the collision gas (Ar) was maintained at 0.2 ml/min. The instrument was operated in the ESI negative mode. Three ion transitions (1 quantifier and 2 qualifier ions) for PEth 16:0/18:1 were recorded in SRM mode: *m/z* 701.4 > 281.2 (quantifier), 701.4 > 255.2, 701.4 > 437.2; PEth-d_5_: *m/z* 706.5 > 281.2 (quantifier), 706.5 > 255.1 and 706.5 > 442.2.

Two decision limits were used for PEth: > 0.050 μmol/l (~35 μg/l) for ‘social drinking’ and >0.30 μmol/l (~210 μg/l) for ‘harmful drinking’ (Helander and Hansson, 2013).

### Analysis of CDT in serum

CDT was determined by a commercial CE- and IVD-marked HPLC method (Chromsystems Instruments & Chemicals GmbH, Gräfelfing, Germany). The decision limit for CDT used to indicate excessive drinking was ≥2.0% disialotransferrin (Helander *et al.*, 2017). The CV value for QC samples was 9.3% for Chromsystems CDT-L1 (mean 1.3%; *n* = 21) and 5.4% for CDT-L2 (mean 2.9%; *n* = 21). The accuracy of the method was further confirmed by comparison with the IFCC CDT HPLC reference method (Helander *et al.*, 2017). Baseline integration of transferrin peaks was used according to the recommendation for this decision limit. In six cases, the chromatograms did not allow for the calculation of the CDT value. These cases were excluded from the study but were all indicated to be CDT negative (˂2.0%).

### Analysis of GGT and MCV and use as combined marker

Measurement of GGT in serum (reference ranges were <1.05 μmol/l·s for males and <0.75 μmol/l·s for females) was made using reagents from Roche Diagnostics, and MCV in whole blood (reference range 85–95 fL) using an instrument from Axon Lab.

A combined GGT/MCV biomarker (both positive) was also used to increase the specificity (Chick *et al.*, 1981; Rosman, 1992).

### Analysis of EtG in serum

EtG analysis was made by an in-house DIN EN ISO 17025 accredited UPLC-MS/MS method, which will be reported in detail separately (Neumann *et al*., 2020). In brief, a 20-μl aliquot of serum was mixed with 200-μl methanol containing 20-pg EtG-d5 (Cerrilliant, Merck KGaA, Darmstadt, Germany). The mixture was shaken thoroughly followed by centrifugation for 10 min at 12,000 × *g*. After centrifugation, 200 μl of the resulting supernatant was transferred to an autosampler vial and evaporated to dryness using heated nitrogen gas. The residue was redissolved in 80-μl 0.1% formic acid and 10 μl was injected into the UPLC. For QC samples at a concentration of 0.50-ng/ml EtG, the CV was 4.4% and bias 2.2%, and at 3.0 ng/ml, the CV was 3.3% and bias 0.3%. The EtG decision limit was 1.0 ng/ml.

### Analysis of ethanol in serum

The serum ethanol concentration was determined using the DRI ethyl alcohol assay from Thermo Fisher Scientific. The QC results for ACQ Science Ethanol 0.50 g/l were mean 0.52, CV 4.4% (*n* = 30); for the 1.30 g/l control mean 1.34, CV 3.4% (*n* = 30) and for the 3.00 g/l control mean 3.04, CV 3.3% (*n* = 38). The ethanol decision limit was 0.10 g/l.

### Statistics

Statistical calculations were done using Xact® chart software (Scilab, Hamburg, Germany) and MedCalc (MedCalc Software, Ostend, Belgium).

## RESULTS

**Fig. 1.**
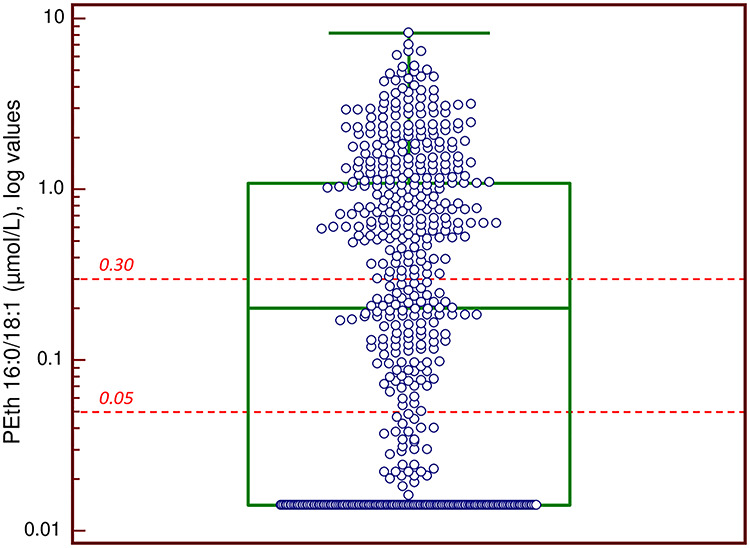
A plot of all phosphatidylethanol (PEth 16:0/18:1) results presented in log scale (*n* = 519). In 155 cases, the PEth measurement was below the LLOQ (0.014 μmol/l). The mean value was 0.79 μmol/l (555 ng/ml), the median value 0.20 μmol/l (140 ng/ml) and the highest value was 8.17 μmol/l (5740 ng/ml). The test for normal distribution was negative. The two reporting limits employed (0.30 μmol/l or ~ 210 μg/l, and 0.05 μmol/l or ~ 35 μg/l, respectively) are indicated (- - -).

A large proportion of the 519 blood specimens tested positive for acute (*n* = 294, 57%) and chronic (*n* = 359, 69%) alcohol biomarkers, demonstrating a high proportion of alcohol drinking in the studied population. PEth in whole blood was the biomarker with overall highest positive rate, with 333 cases (64%) showing a value above the 0.05 μmol/l cut-off (Table 1). PEth was also the only positive chronic biomarker in 100 cases, representing 30% of all PEth-positive cases. For the higher reporting limit indicating ‘risky drinking’ (≥0.30 μmol/l), the number of PEth positives was 238 (46%) and PEth was the only positive chronic biomarker in 38 cases (16%). The distribution of PEth concentrations in all 519 cases is shown in Fig. 1.

**Table 1:** Overview of positive rates for the different biomarkers alone and for test combinations

Alcohol biomarker	Cut-off	*N* (%)
PEth in whole blood	≥0.05 μmol/l	333 (64)
PEth in whole blood	≥0.30 μmol/l	238 (46)
CDT in serum	≥2.0%	126 (24)
GGT in serum	>1.05 μmol/l*s (men), > 0.75 μmol/l*s (women)	216 (42)
MCV in whole blood	>95 fL	136 (41)
GGT and MCV	Both over limits	91 (18)
EtG in serum	≥1.0 ng/ml	294 (57)
Ethanol in serum	≥0.10 g/l	60 (12)
**Combinations**	**PEth (≥0.05) *N* (%)**	**PEth (≥0.3) *N* (%)**
and CDT	126 (24)	114 (22)
and GGT	190 (37)	172 (33)
and MCV	123 (24)	107 (41)
and GGT and MCV	91 (18)	87 (17)
and EtG	278 (54)	225 (43)
and ethanol	60 (12)	56 (11)

**Fig. 2.**
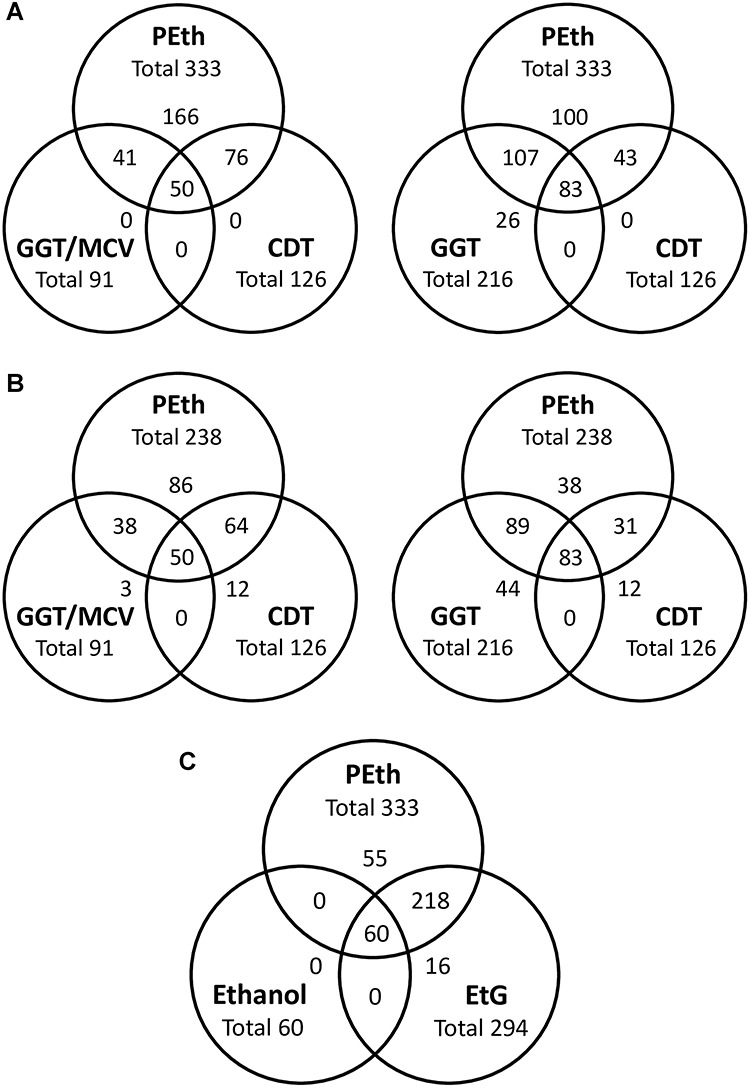
Comparison of the overlap of positive test results between PEth and other tests used as alcohol biomarkers (CDT (% disialotransferrin); GGT; MCV EtG). The total number of positives for each test is given together with the number in each overlapping category, as well as the number of cases not overlapping. (A and C) Applied PEth reporting limit ≥0.05 μmol/l; (B) applied PEth reporting limit ≥0.30 μmol/l.

Compared with PEth, serum CDT and GGT, whole blood MCV and the GGT/MCV combined biomarker were positive in lower numbers of cases (Table 1). GGT was the only positive chronic biomarker in 26 cases, while in no case, MCV or GGT/MCV was the only positive biomarker. A comparison of the test results for chronic alcohol biomarkers, including the two cut-off limits for PEth, is shown in Fig. 2A and B.

The mean (median) serum ethanol concentration among the 60 positive cases was 1.27 (0.95) g/l, respectively. The other acute alcohol biomarker, EtG in serum, was positive in 294 cases (Table 1), with a mean (median) concentration of 915 (134) ng/ml, respectively. A comparison of the test results for PEth with the two acute alcohol biomarkers is presented in Fig. 2C.

**Fig. 3.**
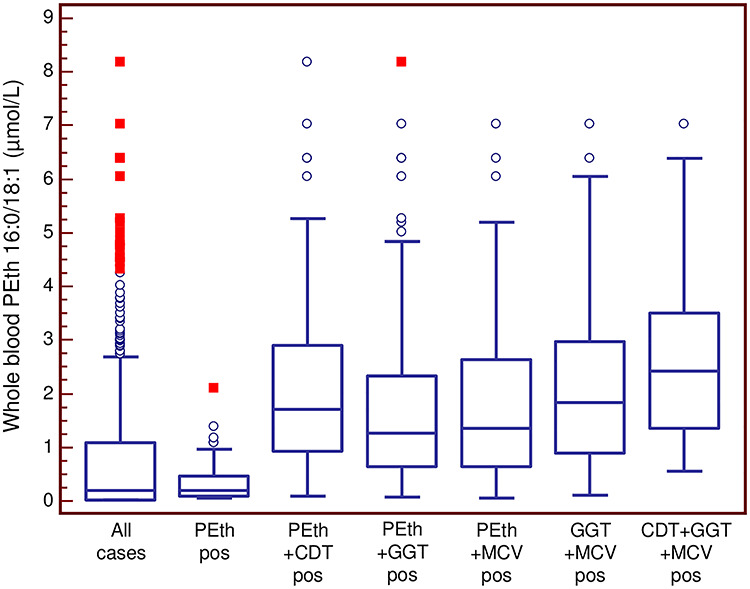
PEth concentrations in cases above 0.05 μmol/l in categories from Fig. 2A. Results are Box-and-whisker plots, where the box represents the 25–75 percentile, the middle line the median, the vertical lines extend to minimum and maximum values, with the exception of statistically ‘outside’ (open circle) and ‘far out’ (filled square) values.

Figure 3 shows the distribution of PEth concentrations in different subgroups from Table 1 and Fig. 2. The highest median concentration was seen in the subgroup being positive for all chronic alcohol biomarkers, followed by the subgroup being positive for PEth and CDT.

### PEth vs CDT

All 126 cases with a positive CDT test were also positive for PEth using the lower (0.050 μmol/l) reporting limit, and in 114 cases (90%) using the higher cut-off (≥0.30 μmol/l). In the CDT-positive cases, the mean and median PEth values were 2.07 and 1.71 μmol/l, respectively, while in the CDT negative cases, the corresponding values were 0.71 and 0.45 μmol/l (*n* = 207). The Mann–Whitney test for independent samples demonstrated a statistically significant difference between the groups (*P* < 0.0001).

A regression analysis for PEth vs CDT concentration values showed a significant correlation (*r* = 0.63, *P* < 0.0001). However, there was a large subpopulation of cases (*n* = 114) where CDT was negative but PEth ≥0.30 μmol/l, and a smaller one where CDT was positive but PEth < 0.30 μmol/l (*n* = 12). In the latter group, the CDT values ranged 2.0–10.1% (median 2.6%) and PEth ranged 0.09–0.28 (median 0.22) μmol/l.

The mean CDT value in the 186 cases with undetected PEth (<0.05 μmol/l) was 1.06 ± 0.29% (SD), while in the subgroup with a negative CDT and a PEth value ≥0.30 μmol/l, the mean CDT value was 1.33 ± 0.35% (*n* = 114). The difference was statistically significant (Mann–Whitney, *P* < 0.0001).

### PEth vs GGT

Of the 333 PEth-positive cases, 190 (57%) were also positive for GGT (Fig. 2A). The PEth concentrations were significantly higher in the GGT-positive cases than in the 143 negative cases, with mean (median) values of 1.71 (1.26) vs 0.57 (0.26) μmol/l, respectively (Mann–Whitney, *P* < 0.0001).

### PEth vs MCV

Of the 333 PEth-positive cases, 123 (37%) were positive for MCV. The PEth concentrations were significantly higher in the MCV-positive cases than in the 210 negative cases, with mean (median) values of 1.77 (1.36) vs 0.90 (0.53) μmol/l, respectively (Mann–Whitney, *P* < 0.0001).

### PEth vs GGT/MCV

Of the 333 PEth-positive cases, 91 (27%) were positive for the GGT/MCV combination (Fig. 2A). The PEth concentrations were significantly higher in the GGT/MCV-positive cases than in the 111 negative cases, with mean (median) values of 2.10 (1.84) vs 0.49 (0.35) μmol/l, respectively (Mann–Whitney, *P* < 0.0001).

### PEth vs EtG

**Fig. 4.**
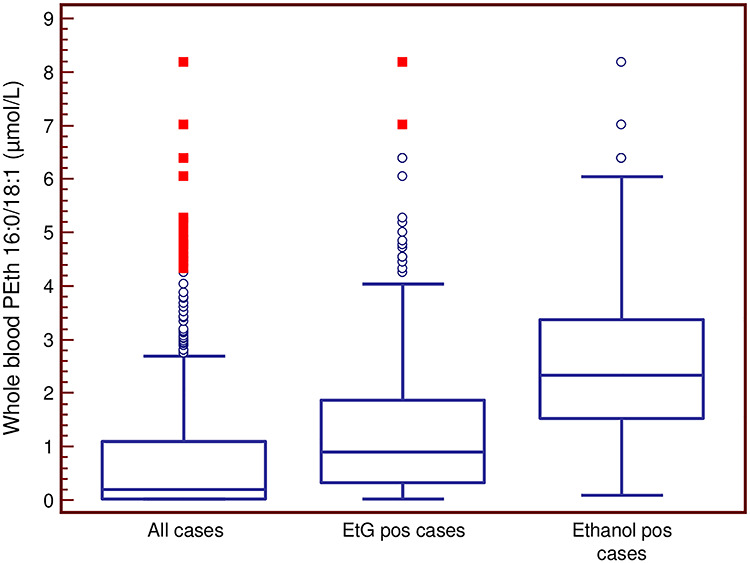
PEth concentrations in cases above 0.05 μmol/l in categories from Fig. 2C. Results are Box-and-whisker plots, where the box represents the 25–75 percentile, the middle line the median, the vertical lines extend to minimum and maximum values, with the exception of statistically ‘outside’ (open circle) and ‘far out’ (filled square) values.

Of the 294 EtG-positive cases, 278 (95%) were also positive for PEth with a mean (median) concentration of 1.41 ± 1.38 (1.00) μmol/l (Fig. 4). Of the 225 EtG-negative cases, 55 (24%) were positive for PEth with the mean (median) value of 0.28 ± 0.34 (0.18) μmol/l.

**Fig. 5.**
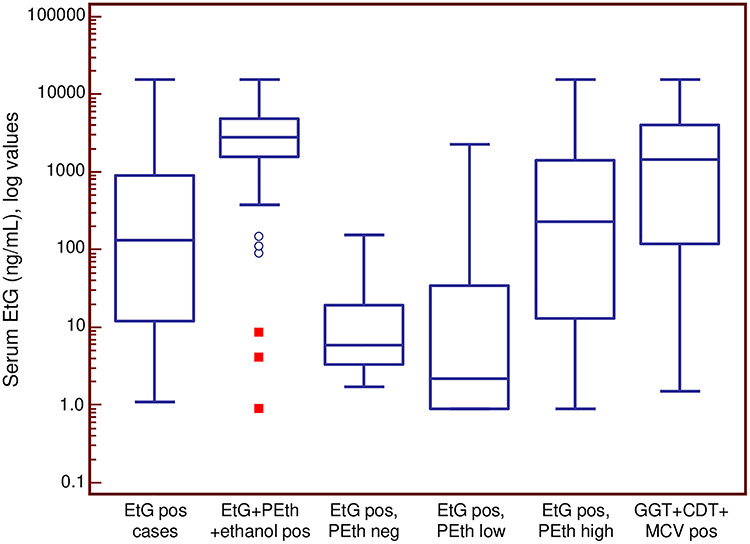
EtG concentrations in cases with different combinations of positive results for PEth and ethanol. Results are Box-and-whisker plots, where the box represents the 25–75 percentile, the middle line the median, the vertical lines extend to minimum and maximum values, with the exception of statistically ‘outside’ (open circle) and ‘far out’ (filled square) values.

Of the 333 PEth-positive cases, 278 (83%) were also positive for EtG, the mean (median) concentration being 966 ± 1826 (178) ng/mL (Fig. 5). Of the 186 PEth-negative cases, 16 (8.6%) were positive for EtG with a mean (median) concentration of 25 ± 46 (6.1) ng/mL. In the 238 cases with a PEth concentration above the upper cut-off (>0.30 μmol/L), 95% were EtG positive, whereas in the 95 cases with PEth concentrations in the range 0.05–0.30 μmol/L, 56% were EtG positive.

### PEth vs ethanol

Of the 60 cases where ethanol was detected, all tested positive for PEth with a mean (median) concentration of 2.65 ± 1.78 (2.32) μmol/L (Fig. 4). Of the 333 PEth-positive cases, 18% were also positive for ethanol (Fig. 2C) and of the 238 cases with a PEth value > 0.30 μmol/L, 25% were positive for ethanol.

### GGT vs GGT/MCV

GGT was positive in more than twice as many cases compared with the GGT/MCV test combination (Table 1). In 125 cases being positive for GGT but negative for MCV, high alcohol consumption was not supported by an elevated PEth or CDT in 26 cases (21%). Among these 26, EtG was detected in 5 cases (19%) but ethanol in no one. In 16 cases, the PEth concentrations ranged 0.05–0.30 μmol/L and EtG was detected in 10 of those (62%), while ethanol was detected in no one. However, in 84 cases high alcohol intake was supported by a positive PEth and/or CDT value. Thus, the addition of MCV as a criterium for a positive GGT test increased test specificity from 80% to 100%, while the sensitivity was reduced to 52%. The PEth concentration difference between the GGT/MCV and GGT positives was statistically different (Mann-Whitney, p = 0.013).

### Ethanol vs EtG

In all 60 ethanol-positive cases, EtG was also detected in serum with a mean (median) concentration of 3392 ± 2637 (2872) ng/mL. In the 234 ethanol-negative cases containing EtG, the mean (median) value was 280 ± 524 (49) ng/mL (Fig. 5).

## DISCUSSION

This study documented a much higher test sensitivity for PEth compared with CDT, GGT and MCV, as indicators of regular high alcohol exposure in subjects undergoing medical investigation at employment agencies and occupational health units. The higher sensitivity of PEth agrees with previous observations (Hartmann *et al.*, 2007; Helander *et al.*, 2012; Kechagias *et al.*, 2015; Walther *et al.*, 2015). Most previous studies comparing PEth with CDT involved patients diagnosed with alcohol dependence (Hartmann *et al.*, 2007; Helander *et al.*, 2012; Walther *et al.*, 2015). In two studies, a total of 200 individuals were monitored over time and in both a two-fold higher test-positive rate was seen for PEth (Helander *et al.*, 2012; Walther *et al.*, 2015), similar to the present observations. In a third study, 56 patients were studied while being in detoxification treatment and, based on the receiver operating characteristic curves, a two-fold higher test sensitivity of PEth over CDT was indicated (Hartmann *et al.*, 2007). In the fourth study, 44 healthy subjects were instructed to drink a fixed amount of red wine daily (33 g/day for men and 16 g/day for women) over a 3-month period (Kechagias *et al.*, 2015). Despite this significant ethanol intake (although not supervised), no subject reached the CDT and PEth cut-offs indicating chronic heavy drinking. However, the statistical analysis demonstrated better performance of PEth in discrimination between the study group and the control group.

An interesting detail from the present study is the observation of 12 cases where CDT was positive but PEth was below the 0.30 μmol/L cut-off used to indicate ‘heavy drinking’, which was contrary to the expected higher test sensitivity of PEth. This could indicate that in some individuals PEth formation in response to alcohol intake is relatively low (Helander *et al.*, 2012), or the result of the slower elimination rate of CDT after stopping drinking alcohol. Nevertheless, in all 12 cases, PEth was detected and when using the interpretation guideline proposed by Ulwelling and Smith (2018), all 12 cases would be classified as ‘significant consumption’.

Reference cut-offs for interpreting PEth concentrations are not firmly established. When the PEth 16:0/18:1 homologue was first used as the single target analyte instead of the sum of all PEth forms, a cut-off concentration of 0.20 μmol/L for heavy drinking was proposed (Zheng *et al.*, 2011). Following a national harmonization of PEth testing in Sweden, the cut-off was later increased to > 0.30 μmol/L (~210 μg/L) to increase test specificity and applied as a generally accepted cut-off to indicate heavy drinking, whereas > 0.05 μmol/L (~35 μg/L) was applied as a lower limit to indicate ‘social drinking’ (Helander and Hansson, 2013). Recently, Ulwelling and Smith (2018) have proposed 20–200 ng/mL (i.e. ~ 0.03–0.30 μmol/L) to indicate ‘significant consumption’ and > 200 ng/mL for ‘heavy consumption’ and Kummer *et al.* (2016) have also favored a similar cut-off (221 ng/mL) for chronic excessive drinking. A recent study from our group also supported the use of 0.30 μmol/L as the cut-off to indicate harmful drinking (>50 g/day) (Helander *et al.*, 2019). This limit is to be considered as safe and allows the interpretation of chronic high alcohol intake even when considering the inter-individual variability in PEth response to alcohol exposure (Helander *et al.*, 2019b).

The CDT test is targeting long-term heavy alcohol consumption, but it is less sensitive than PEth for detecting harmful drinking. This is partly a consequence of the applied CDT cut-off limit of 2.0% recommended for HPLC methods, which is based on the mean + 3SD for controls to increase test specificity (Helander et al., 2017). The subgroup of cases with a PEth concentration > 0.30 μmol/L but a CDT value below 2.0% had significantly higher CDT values than the subgroup with a PEth concentration < 0.30 μmol/L (mean 1.35 vs 1.10%, *P* < 0.0001). This demonstrated that there was indeed a response with increased CDT levels in this PEth positive group. Based on the CDT results for the PEth-negative group, a higher cut-off of 1.9% can be calculated using the mean + 3SD, supporting the recommended cut-off of ≥2.0% for a positive CDT test (Helander *et al.*, 2017).

GGT is a liver function test that is not specific for use as an alcohol biomarker but rather relates to liver damage that may or may not be cause by chronic heavy drinking, while MCV has the feature of high specificity but low sensitivity (Helander and Jones, 2006). In the present study, the use of the GGT/MCV test combination increased the selectivity to 100% but decreased the sensitivity by almost 50%. The observed low sensitivity agrees with previous observations (Chick *et al.*, 1981; Rosman, 1992). Since the PEth and CDT tests covered all cases that were positive by GGT and GGT/MCV, it indicates a very limited value of these tests when PEth and CDT are available. However, GGT may have value as an additional test to reveal alcohol-related liver damage.

An interesting observation was that all cases with a detectable ethanol concentration in blood when visiting the medical units had PEth concentrations above the higher cut-off indicating ‘risky drinking’. For the EtG-positive cases, demonstrating recent alcohol intake but not necessarily being under the influence, there was an association with elevated PEth concentrations. Also the fraction of EtG-positive samples increased with elevated PEth concentrations, from 9% to 95%. The combined use of short- (‘acute’) and long-term (‘chronic’) alcohol biomarkers provide a more complete information regarding drinking habits as noted before (Helander *et al.*, 2012). EtG is usually used as a urinary alcohol biomarker and as a complement to drug testing based on immunoassay screening (Böttcher *et al.*, 2008). This study instead employed EtG measurement in serum by an LC-MS/MS method with a 100-fold lower measuring range. This opens for the possibility to measure both PEth and EtG in the same collected blood sample. EtG measurements might help to differentiate between currently abstinent and drinking patients.

In conclusion, this study confirms the high sensitivity of PEth as a biomarker for alcohol exposure. The inborn high specificity, being an ethanol metabolite, also favors use of this biomarker, although it is not possible to determine the exact amount of alcohol consumed from the test value. GGT and MCV, which have traditionally been employed as alcohol biomarkers, appear to have no additional value over PEth and CDT for detection of chronic high alcohol intake. For optimal detection of harmful drinking in clinical and forensic settings, a combination of short- and long-term alcohol biomarkers covering different amounts of consumption appears to provide the best information regarding individual drinking habits.
